# Stunting, Wasting and Underweight in Sub-Saharan Africa: A Systematic Review

**DOI:** 10.3390/ijerph14080863

**Published:** 2017-08-01

**Authors:** Blessing J. Akombi, Kingsley E. Agho, John J. Hall, Nidhi Wali, Andre M. N. Renzaho, Dafna Merom

**Affiliations:** 1 School of Science and Health, Western Sydney University, Penrith, NSW 2571, Australia; K.Agho@westernsydney.edu.au (K.E.A.); D.Merom@westernsydney.edu.au (D.M.); 2 School of Public Health and Community Medicine, University of New South Wales, Sydney, NSW 2052, Australia; John.Hall@newcastle.edu.au; 3 School of Social Sciences and Psychology, Western Sydney University, Penrith, NSW 2751, Australia; N.Dhingra@westernsydney.edu.au (N.W.); andre.Renzaho@westernsydney.edu.au (A.M.N.R.)

**Keywords:** public health, undernutrition, malnutrition, stunting, wasting, underweight, systematic review, sub-Saharan Africa

## Abstract

*Introduction*: Child undernutrition is a major public health problem. One third of all undernourished children globally reside in Sub-Saharan Africa (SSA). The aim of this study was to systematically review studies to determine the factors associated with stunting, wasting and underweight in SSA and contribute to the existing body of evidence needed for the formulation of effective interventions. *Methods*: This systematic review was conducted using the 2015 Preferred Reporting Items for Systematic reviews and Meta-Analysis (PRISMA) guidelines. Five computerized bibliographic databases were searched: Scopus, PubMed, PsycINFO, CINAHL and Embase. The included studies were rated using eight quality-appraisal criteria derived from the Strengthening the Reporting of Observational studies in Epidemiology (STROBE) checklist: sample size, sampling methodology, response rate, outcome measures, statistical analyses, control for confounding, study limitation, and ethical consideration. *Results*: Of a total of 2810 articles retrieved from the five databases, 49 studies met our inclusion criteria. The most consistent factors associated with childhood stunting, wasting and underweight in SSA were: low mother’s education, increasing child’s age, sex of child (male), wealth index/SES (poor household), prolonged duration of breastfeeding (>12 months), low birth weight, mother’s age (<20 years), source of drinking water (unimproved), low mother’s BMI (<18.5), birth size (small), diarrhoeal episode, low father’s education and place of residence (rural). *Conclusions*: The factors that predispose a child to undernutrition are multisectoral. To yield a sustainable improvement in child nutrition in SSA, a holistic multi-strategy community-based approach is needed that targets the factors associated with undernutrition, thereby setting the region on the path to achieving the WHO global nutrition target by 2025.

## 1. Introduction

The effect of poor nutrition is evident in the suboptimal physical growth of undernourished children, especially in many low and middle income countries [1,2]. Undernutrition in the first 1000 days post-conception is detrimental to the cognitive and physical health of the child as this is the crucial period for proper brain development and linear growth [3]. Undernutrition has both short- and long-term consequences for the health of children, and adversely affects the economic productivity of nations [4]. It is associated with lower educational performance, cognitive deficits and thus poor economic productivity in adulthood; and it creates social and economic challenges in disadvantaged communities [5].

At the conclusion of the millennium development goals (MDG), the proportion of underweight children was reported to have declined globally from 25% in 1990 to 15% in 2015 [6]. However, this decline was not evenly distributed in all regions of the world as nearly 90 percent of all underweight children reside in South East Asia and Sub-Saharan Africa [7]. Also, the number of stunted children had fallen in all regions except sub-Saharan Africa, where the numbers increased by about one third between 1990 and 2013 [7]. According to the 2015 MDG report, SSA accounts for one third of all undernourished children globally with about 39% stunted, 10% wasted and 25% underweight children under-five years of age [6]. These data suggest that despite the global progress that has been achieved, undernutrition remains unacceptably high in SSA, and far from being solved.

Studies on child undernutrition conducted in various individual countries within SSA have highlighted potential causal factors consistent with the UNICEF conceptual framework, which was developed as part of UNICEF Nutrition Strategy for improving child nutrition [8]. As shown in the framework, child malnutrition results from a series of immediate (individual level), underlying (household or family level), and basic (societal level) causes which work in synergy, where determinants at one level influence other levels. The immediate causes are the impact of the basic and underlying causes operating at the individual level through inadequate food intake and disease. The underlying causes focus on household food (in) security, unhealthy household environment, inadequate health services, inadequate care and feeding practices. The basic causes reflects social, cultural, structural, economic and political processes within society which result in inadequate financial, human, physical and social capital that influence household access to adequate quantity and quality of resources. The framework serves as a guide in analyzing the causes of malnutrition and helps in identifying the most appropriate multi-sectoral and multidimensional intervention strategy.

However, no study has collectively and systematically analyzed the most consistent factors associated with child undernutrition across the entire SSA region to drive region-specific interventions which could lead to a decline in undernutrition within the region. Hence, the aim of this study was to review the factors associated with undernutrition in SSA, thus contributing to the existing and growing body of evidence needed to direct effective interventions that prioritize actions which are focused on the immediate, underlying and basic determinants of child undernutrition.

## 2. Methods

### 2.1. Anthropometric Indicators

Three anthropometric indicators were retained for this systematic review: stunting, wasting and underweight. Stunting (Height-for-age) is an indicator of linear growth retardation and cumulative growth deficits in children. Wasting (Weight-for-Height) measures body mass in relation to height and describes current nutritional status. However, underweight (Weight-for-age) is a composite index of height-for-age and weight-for-height. It takes into account both acute malnutrition (wasting) and chronic malnutrition (stunting), but it does not distinguish between the two.

This systematic review focuses on children with a Z-score below minus two standard deviations (−2 SD) from the median of the WHO reference population [9].

### 2.2. Search Strategy

This systematic literature review was conducted using the 2015 Preferred Reporting Items for Systematic reviews and Meta-Analysis (PRISMA) guidelines [10]. A list of relevant MeSH words and sub-headings of keywords was generated and used to comprehensively search peer-reviewed articles from five computerized bibliographic databases: Scopus, PubMed, PsycINFO, CINAHL and Embase. The search covered research conducted in 49 sub-Saharan African countries and published between January 1990 and January 2017. The year 1990 was used as a baseline for this review in order to capture articles published from the inception of the UNICEF conceptual framework on child nutritional status [8] and during the MDG timeline [11]. The articles retrieved from each database were imported into an EndNote library. For additional relevant publications that might have been missed, we searched the bibliographical references of all retrieved articles that met the inclusion criteria, complemented by citation tracking using Google Scholar. The following combination of keywords was used in the search:

- (Malnutr* OR malnourish* OR undernourish* OR undernutr* OR stunt* OR wast* OR underweight*)
- (Child* or under-five* or preschool* or paediatr* or infan* or bab*)
- (Factor* OR determinant* OR correlate* OR cause*)

### 2.3. Inclusion and Exclusion Criteria

Studies were included in the review if they (1) focused on children under-five years; (2) were conducted in SSA; (3) analyzed factors associated with child undernutrition (stunting, wasting and underweight); (4) were published between 1990 and 2017; (5) were cross-sectional studies (qualitative studies, case studies, books, policy briefs or thesis were excluded); (6) were published in a peer-reviewed journal (non-peer reviewed research, review or commentaries were excluded); (7) were written in English.

### 2.4. Data Extraction

All articles identified by the search were exported into an EndNote library and duplicates were removed. The first author (BA) screened all publications by reading the titles and abstracts. In the final screening phase, BA read the full text of the remaining articles and retained studies that met the inclusion/exclusion criteria. All data extraction and appraisals of retrieved studies were independently reviewed by BA and NW, and all disagreements between the two reviewers were resolved through discussion and consensus. The summary of the selected studies were recorded, these included; author, year of publication, country of publication, number of children, age of children, factors associated with undernutrition indicators (stunting, wasting, underweight), and quality assessment score (Table 1). The studies were also grouped by country into SSA sub-regions (West Africa, East Africa, Southern Africa, Central Africa) based on the United Nations (UN) geoscheme classification.

### 2.5. Quality Assessment

Strengthening the reporting of observational studies in Epidemiology (STROBE) checklist [12] was used as a guide to assess the quality of the studies reviewed. The checklist consists of 22 items which are used to evaluate the external validity (based on potential selection bias) and internal validity (based on potential measurement biases and confounding) of cross-sectional studies. After the initial assessment of all reviewed studies based on the 22 STROBE items, the items were further collapsed into 8 quality-appraisal criteria: sample size, sampling methodology, responses rate, outcome measures, statistical analyses, study limitation, ethical consideration and control for confounding as shown in Supplementary Table S1. Scores assigned to each reviewed study range from 0 to 8 points [0 if none of the criteria were met and 8 points if all criteria were met]. The sum of points awarded represented the overall quality score of a study. Studies were rated as poor quality (score ≤ 3); medium quality (4–6); and high quality (≥7).

## 3. Results

**Figure 1:**
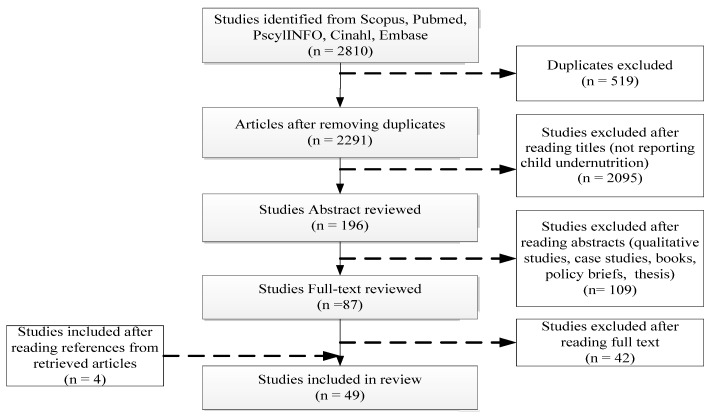
Flow chart for study selection based on PRISMA 2015 guidelines.

A total of 2810 articles were retrieved from the five databases. After the removal of duplicates, 2291 articles were retained. A screening of the titles resulted in the exclusion of 2095 articles. The abstract of the resulting 196 articles were read and screened which led to the exclusion of another 109 articles. The full-text of the remaining 87 articles was reviewed and 42 articles were further excluded. 45 articles met the inclusion criteria. A manual search of the bibliographic references of the retained articles identified additional 4 articles thereby giving a total of 49 studies as shown in Figure 1.

### 3.1. Characteristics of Included Studies

Table 1 shows a summary of the studies included in this review grouped by sub-region. Of the studies conducted; 18 were in West Africa, 23 in East Africa, 4 in Southern Africa and 4 in Central Africa. Sample sizes ranged from 100 to 73,778 participants. Thirty-nine studies reported the factors associated with stunting, 30 studies reported on wasting, 25 studies reported on underweight. The eight criteria used to evaluate the quality of included studies showed that 13 studies (27%) were of high quality, while 31 studies (63%) were of medium quality and 5 studies (10%) were of low quality. The details of domain-specific score are provided in Supplementary Table S2.

In the course of the search, 8 case-control studies and I cohort study were found. Of the case-control studies, 3 were in West Africa [13,14,15] and 4 in East Africa [16,17,18,19]. The only cohort study was conducted in West Africa [20]. However, these studies were not included in the final analysis as our study is focused on cross-sectional studies.

### 3.2. Evidence from Reviewed Studies

The most consistent factors associated with stunting, wasting and underweight as shown in Table 1 were; low mother’s education, increasing child’s age, sex of child (male), wealth index/low SES (poor household), prolonged duration of BF (>12 months), low birth weight, mother’s age (<20 years), source of drinking water (unimproved), low mother’s BMI (<18.5), birth size (small), diarrhoeal episode, low father’s education and place of residence (rural). Other factors also reported were; large family size, geographical region/geopolitical zone, multiple births, short birth interval, high child parity, lack of immunization/vaccination, family type (polygamous), no health care use, lack of health insurance and inappropriate child feeding practices.

In addition to the above mentioned factors, reviewed studies reported fever to be mostly associated with wasting [35,40,41,49] and underweight [21,39,41,47] with only two studies reporting fever as a determinant of stunting [39,40], while only one study conducted in Tanzania associated malaria with wasting [61]. Also, studies reported environmental variables associated with sanitation, hygiene and housing conditions as risk factors for stunting [35,42,58,59,60,64,65], wasting [23,32,37,38,41,58,59] and underweight [39,58,59,60,64,65]. Most studies reported rural residence as a factor associated with undernutrition, but a study conducted in Kenya [52] identified urban residence as a risk factor for wasting and underweight. Prolonged duration of breastfeeding was reported to be mostly associated with stunting [22,23,31,33,58,59,65] and underweight [21,23,52,59,65], with only two studies showing its association with wasting [37,59]. On the other hand, studies reported short duration of breastfeeding to be associated with wasting [46] and underweight [64]. However, a study conducted in Ethiopia identified prolonged duration of breastfeeding to be linked to stunting while short duration of breastfeeding to be associated with wasting.

Child’s age varied across studies [29,36,40,53,59]. However, undernutrition was found in most studies to be associated with increasing child’s age indicating that the older the child, the higher the risk of being stunted, wasted and underweight. Most studies reported male children as having a higher susceptibility to stunting, wasting and underweight. This finding was negated by studies conducted in Kenya [52] and Tanzania [61] which reported female children as being more prone to stunting, wasting and underweight. However, a study conducted in Ethiopia reported male children as being more susceptible to stunting while female to underweight [46]. Geopolitical zone as defined in studies conducted in Nigeria [21,22,29,35] is based on ethnic homogeneity among states with similar cultures, history and close territories as well as political, administrative and commercial cities in Nigeria, this differ from geographical region which are areas broadly classified by physical characteristics. However, these variables are used in describing the physical location of the child and were shown to be mostly associated with stunting [25,27,33,35,41]. Source of drinking water as an environmental factor was categorized into improved and unimproved according to WHO/UNICEF guidelines [70] and was identified as been associated with stunting [41,46,59,66,68], wasting [56,58,59,66] and underweight [41,58,59,66].

## 4. Discussion

In our review, low parent education was reported as one of the consistent factors associated with undernutrition in SSA. A high maternal education translates into greater health care utilization, adoption of modern medical practices and greater female autonomy, which in turn influences health-related decisions that improves child nutritional outcomes [21]. Similarly, a high father’s education also translates to a higher household income and food security. The availability and access to essential food groups that ensure a supply of nutrients is essential in improving child nutrition [71]. The positive relationship between higher educational status and increased wealth index/SES has been reported in studies across SSA [23,29,42]. Households with uneducated parents tend to have low income thus spend less on proper nutrition and are more susceptible to growth failure due to lack of access to sufficient food of adequate quality, poor living conditions, lack of access to basic health care services and greater exposure to diseases. This is the case in SSA where the overall education level is low and poverty level is high.

In this review, low maternal BMI, low birth weight and small birth size were reported to be associated with child undernutrition. Maternal BMI is an important determinant of child undernutrition and is influenced by maternal nutrition, therefore proper nutrition for the mothers during the prenatal and postnatal period is essential in order to improve child growth. The prenatal causes of child suboptimal growth are closely related to maternal undernutrition, and are evident through low maternal BMI which predisposes the foetus to poor growth leading to intrauterine growth retardation; this in turn is strongly associated with small birth size and low birth weight. However, the effect of the prenatal causes of undernutrition can be addressed during the postnatal period through the introduction of appropriate child feeding practises and improved environmental conditions, which if not addressed could predispose a child to the postnatal causes of undernutrition such as low resistance to infection.Child’s age was reported as a consistent factor associated with undernutrition in SSA. Suboptimal growth was observed as the age of the child increased. This finding could be attributed to the challenges of successfully transitioning from exclusive breast-feeding to adequate complementary feeding. Also the increase in child undernutrition with age could be as a result of increased interaction of the older child with the environment which may lead to increased exposure to childhood diseases either through consumption of contaminated foods, drinking water from unimproved sources or poor environmental sanitation.

The double effect of inadequate dietary intake and an unhealthy environment increases a child’s susceptibility to diarrhoeal episode, infections and fever, which in turn depresses appetite, inhibits the absorption of nutrients in food, and increases the need for caloric availability. To maintain an adequate dietary intake, it is crucial that growing children obtain their daily energy from a varied, healthy and balanced diet. The initiation of complementary feeding has to be timely, of good quality and given at the right frequency. Also, improving the quality of health environments through increase in access to safe water supply, proper housing, health services and sanitary facilities for disposing of human waste is important for maintaining a sanitary environment and preventing the spread of illness among children under-5 years especially in rural areas [72].

Prolonged duration of BF for children more than 12 months of age was reported to be associated with undernutrition in SSA. Studies have shown that breast milk alone is nutritionally insufficient for children aged 6 months and over [73,74]. Therefore, for optimal child growth and development, WHO recommends exclusive breastfeeding for the first 6 months of life; thereafter, nutritionally-adequate complementary (solid) foods may be introduced alongside with continued breastfeeding up to 2 years of age and beyond [75]. Prolonged duration of BF with inadequate complementary feeding practices predisposes a child to growth failure due to lack of sufficient nutrients intake needed to fuel their developing brains and bodies [76]. The relationship between undernutrition and prolonged duration of BF is mostly observed among children from poor households and whose parents are uneducated as they are more likely to continue breast-feeding without meeting minimum dietary diversity requirement [74,77]. Therefore, there is a need for education programs targeting mothers with low socioeconomic status which emphasizes the importance of adequate complementary feeding practices in the second half of infancy to be implemented in SSA.

The finding from this review is consistent with that of a systematic review conducted in South Asia that reported similar factors such as suboptimal breastfeeding, inadequate food supply, low household income, illiteracy, unhygienic/substandard living, inappropriate child feeding practice and food insecurity as the correlates of undernutrition [78].In 2015, SSA and South Asia reported the highest prevalence of undernutrition globally [79], which is worsened by the vicious cycle of poverty in these regions [7]. Another systematic review conducted on the determinants of malnutrition in low- and middle-income countries (LMICs) using the INDEPTH health and demographic surveillance systems reported biological causes such as presence of infections and social causes such as wealth, education and urbanization as the factors associated with malnutrition [80].

The identified factors in this review highlight the immediate, underlying and basic causes of child undernutrition as represented in the UNICEF conceptual framework for child nutrition [8]. According to the framework, these factors are interdependent and work in synergy to influence child nutrition. Hence, to effectively address these factors in SSA, global organizations, government, non-governmental organizations (NGOs), private sectors, academia, program managers and society at large need to adopt the comprehensive Framework for Action introduced in 2013 Lancet Maternal and Child Nutrition Series [68] which involves nutrition-specific interventions that directly address the immediate causes of child undernutrition, that is, factors relating to inadequate dietary intake and poor health status; nutrition-sensitive interventions that address the underlying causes, which are associated with household food insecurity, poor quality of care practice for children, and unhealthy living environments; as well as build an enabling environment that addresses the basic causes, more distal factors related to the broad economic, political, environmental, social, and cultural context shaping children’s nutrition [81,82].

This review showed that West Africa and East Africa reported the highest number of studies on child nutrition. This could be a result of the need to address the high level of undernutrition observed within these sub-regions [83]. The demographic, socio-economic and agro-ecological characteristics of these sub-regions have adversely influenced the nutritional status of children as rapid population growth, rising cost of living, desertification, food shortages, unfavorable climatic and drought conditions as well as limited access to land for agricultural purposes has affected food access, availability and production [84,85].

### 4.1. Strengths and Limitations

This systematic review is a comprehensive search of the existing literature on child undernutrition in SSA. Most studies reported were of medium and high quality. However, this study had some limitations. First, qualitative studies were not included in this review as studies selected were restricted to quantitative cross-sectional studies. The inclusion of qualitative studies in systematic reviews enables triangulation of findings or offer alternative explanations [86]. Furthermore, we excluded experimental trial/intervention studies aimed at malnutrition prevention by targeting certain exposures; such studies will provide evidence whether elimination of immediate risks could affect the status of malnutrition but this is outside the scope of this review. Second, this review did not report the pooled estimate for the effect of each factor on undernutrition across all sub-regions; this is due to the fact that the factors were measured differently in each study, thus reporting an estimate for the pool effect would misrepresent the impact of the factors on child nutrition. Third, while this review summarized the individual causes and some of the underlying causes of undernutrition, it does not address the basic or societal causes as well as the effect of genetics, birth defects, and early food allergies or reactions that can cause failure to thrive. Finally, it is worth noting that all studies reviewed did not include any validity assessments of undernutrition; no actual dietary assessments or food insecurity assessments were made or included in the review.

### 4.2. Policy Implications

Findings from this study will enable policy makers and public health researchers to ascertain the most consistent factors associated with undernutrition within countries in SSA for policy actions in the fight to reduce undernutrition in each sub-region in SSA. Such policy actions should focus on educating the female girl, improving the quality of caring practices for children and health environments through the organization of education programs in formal and informal settings within the community, increase in access to safe water supply, sanitation and health care, as well as increase in food security by ensuring adequate availability of nutritious food, especially to people of low socioeconomic status residing in rural areas. This study will also serve as a needs assessment indicator to specific sub-regions having low representation of countries with research on child malnutrition to further explore the factors of child nutrition within its populace.

## 5. Conclusions

The factors associated with undernutrition are multifactorial and interdependent. Hence, there is a need to adopt a multi-strategy community-based approach that targets the immediate, underlying and basic determinants of child undernutrition. Such approach should include counselling sessions for mothers with the aim of improving breastfeeding practices and maternal nutrition, public health campaigns to increase awareness on the importance of proper sanitation and hygiene practices. Further interventions to improve child undernutrition should also focus on cash transfer initiatives to address poverty and increase access to food. These strategies will yield a more sustainable improvement in child nutrition within countries in SSA, thereby setting the region on the path to achieving the WHO global nutrition target by 2025.

## Figures and Tables

**Table 1:** Summary of selected studies.

**Author [Ref.]** **Year, Country**	**No of Children (*N*), Age**	**Factors Associated with Stunting**	**Factors Associated with Wasting**	**Factors Associated with Underweight**	**Quality Assessment (0–8 Points)**
Akombi et al. [21] 2017 *Nigeria*	*N* = 24,529 0–59 months		Place of residence (rural), geopolitical zone (North West), low parent education, home delivery, birth size (small), low mother’s BMI (<18.5), sex (male), No delivery assistance, fever, child’s age (older)	Geographical zone (North West), sex (male), birth size (small), low parent education, home delivery, prolonged duration of BF (>12 months), fever, diarrhoeal episode, low mother’s BMI (<18.5), short birth interval (<24 months)	8 High quality
Akombi et al. [22] 2017 *Nigeria*	*N* = 24,529 0–59 months	Geopolitical zone (North West), diarrhoeal episode, sex (male), birth size (small), wealth index (poor household), prolonged duration of BF (>12 months)			8 High quality
Aheto et al. [23] 2015 *Ghana*	*N* = 2083 0–59 months	Child’s age (older), prolonged duration of BF (>12 months), multiple births, birth size (small), wealth index (poor household), low mother’s BMI (<18.5) and lack of health insurance	Child’s age (older), diarrhoeal episodes, birth size (small), absence of toilet facility, low mother’s BMI (<18.5)	Child’s age (older), prolonged duration of BF (>12 months), multiple births, diarrhoeal episodes, birth size (small), low mother’s education and BMI (<18.5)	6 Medium quality
Ogunlesi et al. [24] 2015 *Nigeria*	*N* = 208 0–59 months		Low mother’s education, Infections, non-exclusive BF		4 Medium quality
Novignon et al. [25] 2015 *Ghana*	*N* = 3675 0–59 months	Place of residence (rural), low mother’s education, wealth index (poor household), sex (male), Geographical region (Northern)	Place of residence (rural), low mother’s education, wealth index (poor household), sex (male), Geographical region (Northern)	Place of residence (rural), low mother’s education, wealth index (poor household), sex (male), Geographical region (Northern)	6 Medium quality
Balogun et al. [26] 2014 *Nigeria*	*N* = 366 2 weeks–59 months	Low father’s education	Diarrhoeal episode	Low father’s education	3 Low quality
Darteh et al. [27] 2014 *Ghana*	*N* = 2379 0–59 months	Sex (male), child’s age (36–47 months), geographical region (Eastern), mother’s age (35–44 years), large family size (5–8 children)			6 Medium quality
Beiersmann et al. [28] 2013 *Burkina Faso*	*N* = 460 6–31 months		Place of residence (rural), child’s age (24–35 months), religion (Muslim), presence of younger siblings		7 High quality
Adekanmbi et al. [29] 2013 *Nigeria*	*N* = 28,647 0–59 months	Sex (male), child’s age (>11 months), multiple birth, low birthweight, low mother’s education, low mother’s BMI (<18.5), wealth index (poor household), short birth interval, geopolitical zone (North West and North East)			8 High quality
Idris et al. [30] 2013 *Nigeria*	*N* = 332 0–59 months	Low mother’s education, large family size (>6 children)	Low mother’s education	Low mother’s education	2 Low quality
Nikoi et al. [31] 2012 *Ghana*	*N* = 2225 0–59 months	Child’s age (older), birth size (small), not vaccinated, prolonged duration of BF (>12 months), low mother’s BMI (<18.5), wealth index (poor household)			6 Medium quality
Olusanya et al. [32] 2010 *Nigeria*	*N* = 5888 0–3 months	Multiple births, child’s age (1–2 months), low mother’s education, mother’s age (<20 years), sex (male)	Multiple births, home delivery, sex (male), child’s age (>30 days), mother’s age (<20 years), shared sanitation facilities	Low mother’s education, mother’s age (>35 or < 20 years), child’s age (>30 days), sex (male), multiple births, home delivery	6 Medium quality
Ellen Van de Poel et al. [33] 2007 *Ghana*	*N* = 3061 0–59 months	Wealth index (poor household), child’s age (older), birth size (small), sex (male), prolonged duration of BF (>12 months), short birth interval, Low mother’s education, no health care use, geographical region (Northern)			6 Medium quality
Odunayo et al. [34] 2006 *Nigeria*	*N* = 420 0–59 months		Overcrowding, low maternal income, type of complementary feeds (infant formula feeds)	Child’s age (24 months)	4 Medium quality
Ukwuani et al. [35] 2003 *Nigeria*	*N* = 5331 0–59 months	Prolonged duration of BF (>12 months), high child parity, toilet facility (pit toilet), wealth index (poor household), sex (male), low mother’s education, low birthweight, no immunization, geopolitical zone (Northern)	Diarrhoeal episode, short duration of BF, fever, low birthweight, religion (Non-Christian), no immunization		6 Medium quality
Ojofeitimi et al. [36] 2003 *Nigeria*	*N* = 230 0–59 months	Low mother’s education, child’s age (12–36 months), high child parity, no immunization, family type (polygamous)	Low mother’s education, child’s age (12–36 months), high child parity, no immunization, family type (polygamous)		3 Low quality
Abidoye et al. [37] 2001 *Nigeria*	*N* = 370 0–59 months		Low mother’s education, marital status (single), non-working mothers, wealth index (poor household), prolonged duration of BF (>12 months), poor water supply and regularity, type of housing and toilet facilities		4 Medium quality
Ighogboja et al. [38] 1992 *Nigeria*	*N* = 400 0–59 months		Wealth index (poor household), family instability, poor environmental sanitation, faulty weaning practices, illiteracy, large family size, infections		5 Medium quality
**East Africa**					
**Author [Ref.]** **Year, Country**	**No of Children (*N*), Age**	**Factors Associated with Stunting**	**Factors Associated with Wasting**	**Factors Associated with Underweight**	**Quality Assessment (0–8 Points)**
Demilew et al. [39] 2017 *Ethiopia*	*N* = 480 24–36 months	Fever, having two children under three years, taking pre-lacteal feeding, early or late initiation of complementary feeding		Fever, lack of latrine utilization, and lack of hand washing practice	8 High quality
Kinyoki et al. [40] 2015 *Somalia*	*N* = 73,778 6–59 months	Fever, diarrhoeal episode, sex (male), child’s age (>12 months), low intensity of vegetation cover	Fever, diarrhoeal episode, sex (male), child’s age (<12 months), low intensity of vegetation cover		6 Medium quality
Yisak et al. [41] 2015 *Ethiopia*	*N* = 791 0–59 months	Geographical region (lowland), high birth order (>6th child), large family size (>2 children), low mother’s BMI (<18.5), source of drinking water (unimproved), place of residence (rural), low mother’s education, sex (male), lacking of farmland, wealth index (poor household)	Sex (male), fever, diarrhoeal episode, no antenatal visit, method of garbage disposal (open field), large family size (>12 children), pre-lacteal feeding	Geographical region (lowland), poor initiation of complementary feeding, diarrhoeal episode, low mother’s BMI (<18.5), high birth order (4–5), home delivery, multiple births, fever, no antenatal visit, mother’s age (<20 years), source of drinking water (unimproved)	7 High quality
Chirande et al. [42] 2015 *Tanzania*	*N* = 7324 0–59 months	Home delivery, no antenatal visit, Low parent education, sex (male), birth size (small), no access to potable drinking water, child’s age (0–23 months), mother’s age (<20 years), low mother’s BMI (<18.5), non-breastfed, wealth index (poor household), place of residence (rural)			8 High quality
Asfaw et al. [43] 2015 *Ethiopia*	*N* = 796 6–59 months	Diarrhoeal episode, sex (male), receiving pre-lacteal feeding at time of birth	Diarrhoeal episode, age at start of complementary feeding (<6 years), lack of family planning	Diarrhoeal episode, sex (male), low father’s education	8 High quality
Fekadu et al. [44] 2015 *Ethiopia*	*N* = 214 6–23 months	Bottle feeding, poor dietary diversity, inappropriate age of complementary feeding initiation	Diarrhoeal episode, bottle feeding	Diarrhoeal episode	7 High quality
Semali et al. [45] 2015 *Tanzania*	*N* = 678 0–59 months	Low mother’s education, father’s age (<35 years), mother’s age (<25 years), no ownership of a mobile phone.			5 Medium quality
Alemayehu et al. [46] 2015 *Ethiopia*	*N* = 605 0–59 months	Low mother’s education, low father’s education, sex (male), source of drinking water (unimproved), Child’s age (12–36 months), large family size (>10 members), late initiation of BF	Late initiation of BF, short duration of BF (6–11 months), mother’s inability to make financial decisions	Late initiation of BF, sex (female), child’s age (12–23 months), lack of toilet facility, type of complementary food (milk), mother’s inability to make financial decisions	7 High quality
Nordang et al. [47] 2015 *Tanzania*	*N* = 152 0–59 months	Increased mother’s work		Diarrhoeal episode, fever, food shortage, dry-season cultivation	8 High quality
Gilbert Habaasa [48] 2015 *Uganda*	*N* = 104 0–59 months	Mother’s occupation (peasant farmers were more likely than pastoralist)		Child’s age (<12 months)	6 Medium quality
Debale et al. [49] 2014 *Ethiopia*	*N* = 9611 0–59 months		Sex (male), birth size (small), child’s age (<11 months), place of residence (rural), no mother’s education, low mother’s BMI (<18.5), wealth index (poor household), diarrhoeal episode, fever		4 Medium quality
Abubakar et al. [50] 2012 *Tanzania*	*N* = 423 1–35 months	Low mother’s education, child’s age (older) concerns over child growth and development		Concerns over child’s growth and development, proximity to water source	5 Medium quality
Abuya et al. [51] 2012 *Kenya*	*N* = 5156 0–42 months	Low mother’s education, low birth weight, sex (male), marital status (single), high mother’s parity (1 < birth), home delivery, low SES			6 Medium quality
Gewa et al. [52] 2011 *Kenya*	*N* = 3793 0–59 months	Sex (female), birth size (small), prolonged duration of BF(>12 months), short maternal stature (<150 cm), maternal underweight, low mother’s education, wealth index (poor household)	Sex (female), diarrhoeal episode, maternal underweight, low mother’s education, wealth index (poor household), place of residence (urban)	Sex (female), birth size (small), diarrhoeal episode, prolonged duration of BF (>12 months), maternal underweight, low mother’s education, wealth index (poor household), place of residence (urban)	7 High quality
Mulugeta et al. [53] 2010 *Ethiopia*	*N* = 318 0–59 months	Child’s age (>6 months), low mother’s height, inadequate complementary foods, geographical region (Eastern and central)	Child’s age (>6 months)	Child’s age (>6 months), low mother’s weight, inadequate complementary foods	4 Medium quality
Engebretsen et al. [54] 2008 *Uganda*	*N* = 723 0–11 months	Child’s age (older), sex (male), wealth index (poor household), sub-optimal infant feeding practices after birth	Diarrhoeal episode		6 Medium quality
Nyaruhucha et al. [55] 2006 *Tanzania*	*N* = 250 0–59 months			Low mother’s education, mother’s age (<20 years), child’s age (>12 months), large family size (>7 members), prolonged duration of food shortage, marital status (married polygamous)	3 Low quality
Bloss et al. [56] 2004 *Kenya*	*N* = 175 0–59 months	Child’s age (>12 months), immunization not up-to-date	Diarrhoeal episode, early initiation of complementary feeding (<6 months), source of drinking water (unimproved), immunization not up-to-date, no kitchen garden	Child’s age (>12 months), early initiation of complementary feeding (<6 months), upper respiratory infection or other illness	4 Medium quality
Wamani et al. [57] 2004 *Uganda*	*N* = 720 0–23 months	Low mother’s education, sex (male), child’s age (older), wealth index (poor household)			7 High quality
Kikafunda et al. [58] 1998 *Uganda*	*N* = 261 0–59 months	Child’s age (older), poor health status, prolonged duration of BF (>18 months), low SES, low mother’s education, lack of paraffin as fuel, consumption of low energy density food, presence of eye pathology, consumption of small meals	Place of residence (rural), poor health status, source of drinking water (unimproved), lack of charcoal as fuel, lack of milk consumption, lack of personal hygiene	Place of residence (rural), poor health status, source of drinking water (unimproved), lack of charcoal as fuel, lack of milk consumption, lack of personal hygiene	4 Medium quality
Getaneh et al. [59] 1998 *Ethiopia*	*N* = 669 0–59 months	Child’s age (>2 years), low SES, poor housing condition, non-availability of latrine, source of drinking water (unimproved), an attack of pertussis, not completing immunization, prolonged duration of BF (>12 months), nutritionally inadequate diet	Child’s age (>2 years), low SES, non-availability of latrine, source of drinking water (unimproved), an attack of pertussis, not completing immunization, nutritionally inadequate diet, poor housing condition, prolonged duration of BF (>12 months)	Child’s age (>2 years), low SES, poor housing condition, non-availability of latrine, source of drinking water (unimproved), an attack of pertussis, not completing immunization, prolonged duration of BF (>12 months), nutritionally inadequate diet	6 Medium quality
Vella et al. [60] 1995 *Uganda*	*N* = 4320 0–59 months	Diarrhoeal episode, non-availability of latrine, low father’s education, crowded household, pregnant mother, not being breastfed, religion (Muslim)	Diarrhoeal episode, marital status (divorced), religion (Muslim or Catholic), distance from health centre (>4 miles)	Diarrhoeal episode, marital status (divorced), pregnant mother, non-availability of latrine, large family size (>3 members), distance from health centre (>4 miles), father’s occupation (subsistence farmer), low father’s education, birth order (first or second child), religion (Catholic)	4 Medium quality
Mbago et al. [61] 1992 *Tanzania*	*N* = 949 12–48 months		Sex (female), place of residence (small towns), malaria	Sex (female), low mother’s education, mother’s age (<25 years), diarrhoeal episode, low frequency of feeding	3 Low quality
**Southern Africa**					
**Author [Ref.]** **Year, Country**	**No of Children (*N*), Age**	**Factors Associated with Stunting**	**Factors Associated with Wasting**	**Factors Associated with Underweight**	**Quality Assessment (0–8 Points)**
Lesiapeto et al. [62] 2010 *South Africa*	*N* = 2485 0–59 months	Sex (male), mother’s perception that child is not growing well		Sex (male), low mother’s education, mother’s perception that child is not growing well, currently not BF (for children <24 months), diarrhoeal episode, irregular source of income	6 Medium quality
Willey et al. [63] 2009 *South Africa*	*N* = 1186 0–30 months	Sex (male), low birth weight, mother’s age (<20 years), unemployed mothers, ethnicity (black)			4 Medium quality
Chopra et al. [64] 2003 *South Africa*	*N* = 868 0–35 months	Low mother’s education, absence of toilet facility, low birth weight, house made of traditional materials, long distance from health clinic (>1 h)		Absence of father, low mother’s education, absence of toilet facility, short duration of BF (<1 month), low birth weight, house made of traditional materials	4 Medium quality
Tharakan et al. [65] 1999 *Botswana*	*N* = 734 0–59 months	Child’s age (>12 months), low birth weight, prolonged duration of BF (>12 months), place of residence (rural), toilet facility (pit latrine), low mother’s education, low father’s education	Low birth weight, gender of family head (female), toilet facility (pit latrine), diarrhoeal episode	Place of residence (rural), gender of family head (female), low birth weight, prolonged duration of BF (>12 months), child’s age (>12 months), toilet facility (pit latrine), low mother’s education, low father’s education	4 Medium quality
**Central Africa**					
**Author [Ref.]** **Year, Country**	**No of Children (*N*), Age**	**Factors Associated with Stunting**	**Factors Associated with Wasting**	**Factors Associated with Underweight**	**Quality Assessment (0–8 points)**
Nagahori et al. [66] 2015 *Cameroun*	*N* = 100 5–24 months	Mother’s age (<20 years), child’s age (older), low mother’s education, mothers with no family planning information, source of drinking water (unimproved)	Mother’s age (low), low mother’s education, mothers with no family planning information, source of drinking water (unimproved)	Mother’s age (low), low mother’s education, mothers with no family planning information, source of drinking water (unimproved)	6 Medium quality
Nolla et al. [67] 2014 *Cameroun*	*N* = 475 2 weeks–59 months	Low mother’s education, low fruits and vegetables intake	Low mother’s education, , low fruits and vegetables intake	Low mother’s education, , low fruits and vegetables intake	6 Medium quality
Mukatay et al. [68] 2010 *DRC*	*N* = 1963 0–59 months	Low mother’s education, source of drinking water (unimproved), sex (male), child’s age (>11 months)	Decreased appetite, diarrhoeal episode, child’s age (<12 months)		4 Medium quality
Delpeuch et al. [69] 1999 *DRC*	*N* = 2373 0–59 months	Low SES, low mother’s education, place of residence (peripheral area)	Child’s age (12–23 months)		4 Medium quality

## Supplementary Materials

The following are available online at www.mdpi.com/1660-4601/14/8/863/s1, Table S1: Assessment tools used for the quality appraisal of the included studies, based on STROBE checklist; Table S2: Quality assessment of included studies.
