# Supplementary material for: Stunting, Wasting and Underweight in Sub-Saharan Africa: A Systematic Review
Source: Int J Environ Res Public Health. 2017 Aug 1;14(8):863. doi: 10.3390/ijerph14080863 (PMC5580567; doi:10.3390/ijerph14080863)
Supplement: Supplementary file 1 [file ijerph-14-00863-s001.zip › Supplementary Tables/Supplementary table 1.pdf]

Supplementary table 1: Assessment tools used for the quality appraisal of the included studies, based on STROBE checklist.

**Sample size**	**Sampling methodology (Representativeness)**	**Responses rate**	**Outcome measure**	**Statistical analysis**	**Confounding**	**Study limitations**	**Ethical considerations**
[1 point] Study reported sample size calculation	[1 point] Random selection from representative list of the target population	[1 point] High response rate (> 70%)	[1 point] Valid acceptable definition of malnutrition (stunting, wasting, underweight) based on either WHO growth standards or NCHS* and objectively measured	[1 point] Statistical techniques and reasons for choosing techniques are fully explained.	[1 point] Well adjusted (Individual, household and community level factors). Adjusted and unadjusted Odd ratio (OR) or Relative ratio (RR), confidence interval and *p*-values were well presented	[1 point] Stated study limitations	1 [point] Studies with ethical consideration
[0 point] No sample size calculation	[0 point] Non- random selection but from sampling frame that represent the community	[0 point] Moderate/Low response rate (<70%)	[0 point] No valid definition of malnutrition	[0 point] Statistical techniques are not explained.	[0 point] Minimal adjusted for some or none of the confounders.	[0 point] No stated study limitations	[0 point] Studies with no ethical consideration

*NCHS = National centre for health statistics
