# Supplementary material for: Stunting, Wasting and Underweight in Sub-Saharan Africa: A Systematic Review
Source: Int J Environ Res Public Health. 2017 Aug 1;14(8):863. doi: 10.3390/ijerph14080863 (PMC5580567; doi:10.3390/ijerph14080863)
Supplement: Supplementary file 1 [file ijerph-14-00863-s001.zip › Supplementary Tables/Supplementary table 2.pdf]

Supplementary table 2: Quality assessment of included studies

Author, Year Country	Sample size	Sampling methodology (Representativeness)	Response rate	Outcome measure	Statistical analysis	Confounding	Study limitations	Ethical considerations	Quality assessment Score
**WEST AFRICA**									
Akombi *et al*. 2017 Nigeria	1	1	1	1	1	1	1	1	8
Akombi *et al*. 2017 Nigeria	1	1	1	1	1	1	1	1	8
Aheto *et al*. 2015 Ghana	1	1	0	1	1	1	1	0	6
Ogunlesi *et al*. 2015 Nigeria	0	1	0	1	0	1	1	0	4
Novignon *et al*. 2015 Ghana	1	1	0	1	1	1	1	0	6
Balogun *et al*. 2014 Nigeria	0	1	0	0	0	1	0	1	3
Darteh *et al*. 2014 Ghana	1	0	1	1	1	1	1	0	6
Beiersmann *et al*. 2013 Burkina Faso	1	1	0	1	1	1	1	1	7
Adekanmbi *et al*. 2013 Nigeria	1	1	1	1	1	1	1	1	8
Idris *et al*. 2013 Nigeria	0	0	0	0	0	1	0	1	2
Nikoi *et al*. 2012 Ghana	1	1	1	1	1	0	1	0	6
Olusanya *et al*. 2010 Nigeria	0	1	0	1	1	1	1	1	6
Ellen Van de Poel *et al*. 2007 Ghana	1	1	0	1	1	1	1	0	6

Odunayo *et al*. 2006 Nigeria	0	1	0	1	1	1	0	0	4
Ukwuani *et al*. 2003 Nigeria	1	1	0	1	1	1	1	0	6
Ojofeitimi *et al*. 2003 Nigeria	1	1	0	0	0	1	0	0	3
Abidoye *et al*. 2000 Nigeria	0	1	0	1	1	1	0	0	4
Ighogboja *et al*. 1992 Nigeria	0	1	0	1	1	1	1	0	5
**EAST AFRICA**									
Demilew *et al*. 2017 Ethiopia	1	1	1	1	1	1	1	1	8
Kinyoki *et al*. 2015 Somalia	0	1	0	1	1	1	1	1	6
Yisak *et al*. 2015 Ethiopia	1	1	0	1	1	1	1	1	7
Chirande *et al*. 2015 Tanzania	1	1	1	1	1	1	1	1	8
Asfaw *et al*. 2015 Ethiopia	1	1	1	1	1	1	1	1	8
Fekadu *et al*. 2015 Ethiopia	1	1	1	0	1	1	1	1	7
Semali *et al*. 2015 Tanzania	1	1	0	1	1	1	0	1	5
Alemayehu *et al*. 2015 Ethiopia	1	1	1	1	0	1	1	1	7
Nordang *et al*. 2015 Tanzania	1	1	1	1	1	1	1	1	8
Gilbert Habaasa 2015 Uganda	0	1	0	1	1	1	1	1	6

Debale *et al.* 2014 Ethiopia	0	1	0	0	1	1	1	0	4
Abubakar *et al.* 2012 Tanzania	0	1	0	1	1	1	0	1	5
Abuya *et al.* 2012 Kenya	0	1	0	1	1	1	1	1	6
Gewa *et al.* 2011 Kenya	1	1	1	1	1	1	1	0	7
Mulugeta *et al.* 2010 Ethiopia	0	1	0	1	0	1	0	1	4
Engebretsen *et al.* 2008 Uganda	0	1	0	1	1	1	1	1	6
Nyaruhucha *et al.* 2006 Tanzania	1	1	0	0	0	1	0	0	3
Bloss *et al.* 2004 Kenya	0	0	0	1	1	1	1	0	4
Wamani *et al.* 2004 Uganda	1	1	0	1	1	1	1	1	7
Kikafunda *et al.* 1998 Uganda	0	1	0	1	1	1	0	0	4
Getaneh *et al.* 1998 Ethiopia	0	1	0	1	1	1	1	1	6
Vella *et al.* 1995 Uganda	0	1	0	1	1	1	0	0	4
Mbago *et al.* 1992 Tanzania	0	1	0	0	1	1	0	0	3
**SOUTHERN AFRICA**									
Lesiapeto *et al.* 2010 South Africa	0	1	0	1	1	1	1	1	6

Willey *et al.* 2009<br>South Africa	0	0	0	1	1	1	0	1	4
Chopra *et al.* 2003<br>South Africa	0	1	0	1	1	1	0	0	4
Tharakan *et al.* 1999<br>Botswana	0	1	0	1	1	1	0	0	4

CENTRAL AFRICA

Nagahori *et al.* 2015<br>Cameroun	0	1	0	1	1	1	1	1	6
Nolla *et al.* 2014<br>Cameroun	1	1	0	1	1	0	1	1	6
Mukatay *et al.* 2010<br>DRC*	0	0	0	1	1	1	0	1	4
Delpeuch *et al.* 1999<br>DRC*	0	1	0	1	1	1	0	0	4

*DRC = Democratic Republic of Congo
